# SMYD3 promotes endometrial cancer through epigenetic regulation of LIG4/XRCC4/XLF complex in non-homologous end joining repair

**DOI:** 10.1038/s41389-023-00503-0

**Published:** 2024-01-08

**Authors:** Yujia Huang, Ming Tang, Zhiyi Hu, Bailian Cai, Guofang Chen, Lijun Jiang, Yan Xia, Pujun Guan, Xiaoqi Li, Zhiyong Mao, Xiaoping Wan, Wen Lu

**Affiliations:** 1 Shanghai Key Laboratory of Maternal Fetal Medicine, Shanghai Institute of Maternal-Fetal Medicine and Gynecologic Oncology, Clinical and Translational Research Center, Shanghai First Maternity and Infant Hospital, School of Medicine, Tongji University, Shanghai, 200092 China; 2 Department of Radiology, West China Hospital, Sichuan University, Chengdu, 610000 China; 3 Department of Biochemistry and Molecular Biology, University of Nebraska Medical Center, Omaha, NE USA

**Keywords:** Cancer therapy, Endometrial cancer

## Abstract

Endometrial cancer (EC) stands as one of the most prevalent malignancies affecting the female genital tract, witnessing a rapid surge in incidence globally. Despite the well-established association of histone methyltransferase SMYD3 with the development and progression of various cancers, its specific oncogenic role in endometrial cancer remains unexplored. In the present study, we report that the expression level of SMYD3 is significantly upregulated in EC samples and associated with EC progression. Through meticulous in vivo and in vitro experiments, we reveal that depletion of SMYD3 curtails cell proliferation, migration, and invasion capabilities, leading to compromised non-homologous end joining repair (NHEJ) and heightened sensitivity of EC cells to radiation. Furthermore, our pathway enrichment analysis underscores the pivotal involvement of the DNA damage repair pathway in regulating EC progression. Mechanistically, in response to DNA damage, SMYD3 is recruited to these sites in a PARP1-dependent manner, specifically methylating LIG4. This methylation sets off a sequential assembly of the LIG4/XRCC4/XLF complex, actively participating in the NHEJ pathway and thereby fostering EC progression. Notably, our findings highlight the promise of SMYD3 as a crucial player in NHEJ repair and its direct correlation with EC progression. Intriguingly, pharmacological intervention targeting SMYD3 with its specific inhibitor, BCI-121, emerges as a potent strategy, markedly suppressing the tumorigenicity of EC cells and significantly enhancing the efficacy of radiotherapy. Collectively, our comprehensive data position SMYD3 as a central factor in NHEJ repair and underscore its potential as a promising pharmacological target for endometrial cancer therapy, validated through both in vitro and in vivo systems.

## Introduction

Endometrial cancer stands as the most prevalent gynecological cancer among women, with its incidence and new diagnoses escalating rapidly on a global scale. Despite this surge, the 5-year survival rate has not shown significant improvement in recent years [1–3], underscoring the limitations of existing clinical treatments. Recognizing the imperative need for enhanced therapeutic strategies, a profound comprehension of the developmental mechanisms underlying endometrial cancer becomes pivotal. Such understanding serves as a foundation for identifying novel targets in the pursuit of more effective treatments for endometrial cancer.

Previous large-scale sequencing studies on primary endometrial cancer samples have consistently highlighted the involvement of DNA repair mechanisms in both the tumorigenesis and progression of endometrial cancer [4, 5]. Notably, this association has led to the prioritization of DNA repair for further investigation in clinical trials for endometrial cancer, as approved by The US National Cancer Institute [6]. DNA repair serves as a highly effective mechanism for cells to uphold genome integrity in the face of various endogenous [7] and exogenous stressors [8]. Proper and efficient repair of DNA damage is crucial, as inadequate repair compromises genome integrity, resulting in the accumulation of DNA damage. Persistent DNA damage, in turn, diminishes genome stability and fosters conditions conducive to cancer development [9]. However, it is noteworthy that targeting genes associated with the DNA damage response presents a promising strategy in cancer treatment, especially when combined with chemotherapy or irradiation. These therapies are designed to kill cancer cells mostly by inducing toxic DNA damage. Therefore, targeting proteins related to DNA damage repair has been explored as an attractive therapeutic target [9].

According to previous research findings, post-translational modifications, encompassing phosphorylation, ubiquitylation, acetylation, SUMOylation, PARylation, and methylation, play pivotal roles in modulating the DNA damage repair process. These modifications exert their influence by impacting the expression or recruitment of DNA damage response (DDR) factors and by altering the dynamic status of chromatin [10–14]. Methylation, dynamically regulated by methyltransferases and demethylases, also plays a vital role in DNA damage and repair. For histone methylation, H3K4, H3K9, H3K27, H3K36, H3K79, and H4K20 have all been found to be related to DNA damage-mediated methylation dynamics [15–19]. Non-histone methylation also plays a crucial role, with studies demonstrating that the methylation of MRE11 and 53BP1 enhances their DNA binding abilities, promoting their focal accumulation at Double-Strand Break (DSB) sites [20–22]. Additionally, the methylation of DNA ligase 1 by G9a facilitates the recruitment of DNMT1 to methylate newly synthesized DNA at replication forks [12]. Although an increasing number of studies have provided insights into the function of methylation in the DNA damage response, the protein methylation modification in regulating the NHEJ repair pathway remains largely unknown.

The SET and MYND domain-containing protein 3 (SMYD3) belongs to the SMYD lysine methyltransferase family and actively participates in the methylation of a diverse range of substrates, both histone and non-histone [23]. The SET domain within SMYD proteins serves as the catalyst for lysine methylation, while the MYND domain, characterized by a cysteine-rich zinc finger motif, is instrumental in facilitating protein‒protein interactions [24]. SMYD3 assumes a crucial role in tumorigenesis [25], as evidenced by its consistently elevated expression during the initiation and progression of various tumor types, including colorectal cancer, hepatocellular cancer, breast cancer, and ovarian cancer [26–28]. This widespread occurrence suggests its pivotal involvement in tumor initiation and progression. In addition to its association with tumorigenesis, SMYD3 has also been reported to methylate histone H2A.Z.1 at lysine 101 in the promoter of cyclin A1, promoting cyclin A1 transcription in breast cancer [29]. Furthermore, SMYD3-mediated methylation of MAP3K2 has been identified as a promoter of RAS-driven carcinoma formation by activating the RAS-ERK signaling pathway [30]. Despite these substantial findings across various tumor types, the specific role of SMYD3 in endometrial cancer remains elusive and has yet to be elucidated.

Based on previous research, DNA double-strand breaks (DSBs) represent the most severe form of DNA damage, initiating intricate cellular processes [9]. Mammalian cells employ two primary pathways for DSB repair: homologous recombination (HR) repair and non-homologous end-joining repair (NHEJ). HR is an error-free pathway that utilizes an intact sister chromatid as a template in S/G2 phases [31]. NHEJ, on the contrary, is a rapid, high-capacity pathway that joins two DNA ends with no presence of an intact template through the whole cell cycle [32]. The initiation of NHEJ involves the binding of the KU70-KU80 heterodimer to DSB ends, followed by the recruitment of the DNA-dependent protein kinase catalytic subunit (DNA-PKcs) to chromatin, ultimately establishing a long-range synapse with the KU70-KU80 complex [32, 33]. This process aligns the two DNA ends closely, requiring the DNA ligase IV (LIG4)/XRCC4/XRCC4-like factor (XLF) complex for effective ligation [32, 33]. LIG4 serves as a primary ligation factor, with XRCC4 contributing to the stability and activity of LIG4 [34]. XLF, in turn, enhances the ligation activity of LIG4/XRCC4 [35]. It has been reported that SMYD3 is involved in HR repair by promoting the translocation of RAD51 to DNA damage sites. Co-targeting SMYD3/PARP leads to synthetic lethality in HR-proficient cancer cells [36]. However, whether SMYD3 is involved in NHEJ repair and participates in endometrial cancer development remains unknown.

In this study, we report for the first time that SMYD3 plays a significant role in endometrial cancer progression, and we identified a novel mechanism of SMYD3-mediated NHEJ repair in endometrial cancer. In detail, we provide evidence that SMYD3 expression is upregulated in endometrial cancer and associated with EC progression. Knockdown of SMYD3 inhibits the proliferation, migration, and invasion abilities of endometrial cancer cells in vivo and in vitro. By conducting pathway analysis in the TCGA database, we found that the DNA repair pathway is involved in endometrial cancer progression. In response to DNA damage, SMYD3 is quickly recruited to DNA damage sites in a PARP1-dependent manner and methylates LIG4, which affects the recruitment of the LIG4/XRCC4/XLF complex during NHEJ repair. More importantly, the inhibition of SMYD3 with its specific inhibitor BCI-121 significantly sensitized endometrial cancer cells to radiotherapy. Our results suggest that SMYD3 might be a promising therapeutic target for endometrial cancer.

## Materials and methods

### Cell culture

HEC1B and ISK cell lines were cultured in DMEM/F-12 (L310KJ, Basal Media) supplemented with 10% FBS (04-001-1 A, Biological Industries) and 1% penicillin/streptomycin (15140-122, Gibco). HEK-293FT, U2OS and EJ5-U2OS cells lines were cultured in DMEM (L110KJ, Basal Media) supplemented with 10% FBS (04-001-1 A, Biological Industries) and 1% penicillin/streptomycin (15140-122, Gibco). All cell lines were incubated in a 5% CO_2_ humidified incubator (Thermo Fisher Heracell 240i, Thermo Fisher Scientific) at 37 °C. Mycoplasma testing was conducted regularly.

### Plasmids, shRNA, and siRNA

The pLVX-EF1α-IRES-Puro-SMYD3-3×FLAG was constructed by General Biol (Anhui, China) and used as the template of SMYD3. Site-directed mutagenesis was performed using KOD Kit (NEB) according to the manufacturer’s instructions. The GFP-SMYD3 was subcloned into pEGFP-C2 backbone. The ORF of human XLF was amplified from cDNA of HCA2-hTERT cells and subsequently cloned into pEGFP-C2 backbone. The GFP-LIG4, GFP-XRCC4 were kindly given by professor Zhiyong Mao from Tongji University School of Medicine. The shRNAs targeting SMYD3 were subcloned into the pLKO.1 plasmid (Addgene, USA). The viruses were collected from the medium after 72 h transfection. For knockdown experiments, cells were infected with viruses 24 h in the presence of polybrene (3 mg/ml, HY-112735, MedChemExpress). The siRNAs targeting SMYD3 were synthesized by GenePharma (Shanghai, China). Scrambled siRNA was used as negative control. shRNAs used in this study were as follows:

shRFP targeted sequence: 5’-GCTCCGTGAACGGCCACGAGT-3’;

shSMYD3-1 targeted sequence: 5’-GAACGCAGTCAGAGGGAAATA-3’;

shSMYD3-2 targeted sequence: 5’-GCTTCCCGATATCAACATCTA-3’;

shPARP1 targeted sequence: 5’-TGGAAAGATGTTAAGCATTTA-3’.

siRNAs used in this study were as follows:

siCONTROL targeted sequence: 5’-GCGUUGCUCGGAUCAGAAA-3’

siSMYD3-1 targeted sequence: 5’-GAUUGAAGAUUUGAUUCUA-3’;

siSMYD3-2 targeted sequence: 5’-UCACAGCUGUGACCCCAAC-3’.

### RNA extraction and qPCR

Total RNAs were extracted using TRIzol reagent (Invitrogen, USA). mRNA expression was detected by Hieff qPCR SYBR green master mix (Yeasen, China) on an ABI Prism 700 thermal cycler (Applied Biosystems, USA). All the primers were purchased from General Biol (Anhui, China). Primers used in this study were as follows:

SMYD3-1-forward: GCGCCCCGGAGAGCTA;

SMYD3-1-reverse: GCATCAGCTTTTCCTTCCCGA;

SMYD3-2-forward: GCCGTGACCCCGCTG;

SMYD3-2-reverse: CATCAGCTTTTCCTTCCCGA;

GAPDH-forward: TCCTGTTCGACAGTCAGCCGCA;

GAPDH-reverse: ACCAGGCGCCCAATACGACCA.

### Antibodies

SMYD3 (12011-1-AP, Proteintech), LIG4 (A1743, Abclonal), XRCC4 (A7539, Abclonal), XLF (A4985, Abclonal), Anti-Methylated Lysine (mono and di-methyl) (ab23366, Abcam), H3 (ab1791, Abcam), γH2AX (9718 S, Cell Signaling Technology), DNA-PKcs (ab44815, Abcam), KU70 (A0883, Abclonal), KU80 (A5862, Abclonal), GFP (2956 T, Cell Signaling Technology), GFP (50430-2-AP, Proteintech), FLAG (M185-7, MBL), H3K4me2 (9725, CST), H3K4me3 (9751, CST), TUBULIN (AP0064, Bioworld), GAPDH-HRP (60004, Proteintech), Goat anti-Rabbit secondary antibody (A32733, Invitrogen).

### Laser micro-irradiation-coupled live-cell imaging

After transfected with plasmids for 24 h, cells were presensitized with 10 μM BrdU (5911439, BioGems) for 16 h at 37 °C. Then, laser micro-irradiation was performed using a confocal microscope (TCS SP8, Leica) coupled with the Micropoint system (Andor Technology). The nucleus of the cells was locally irradiated with a 365 nm UV laser from the Micropoint system to form DNA damage. Green fluorescence would be seen if ectopic protein accumulates at the DNA damage sites. The fluorescence intensity of GFP signals at laser micro-irradiated sites was measured using ImageJ software.

### SDS-PAGE and Western blotting

Total cell lysates were collected in RIPA lysis buffer. Western blotting analysis was performed as described. Briefly, equal numbers of cells were washed with PBS twice. The cell pellet was re-suspended in the same amount of loading buffer and boiled for 10 min. Equal amounts of protein were loaded on a 7.5-12.5% SDS-polyacrylamide electrophoresis gel.

### Chromatin fractionation

Cells were harvested and washed with PBS twice. The cell pellet was resuspended in buffer I [37] for 3 min on ice and centrifuged at 13,000 rpm at 4 °C for 3 min. The insoluble pellet containing the chromatin sample was washed in PBS twice by centrifugation at 13,000 rpm at 4 °C for 3 min. Re-suspended the remaining pellet in SDS loading buffer and boiled for 10 min before Western blotting assay.

### Coimmunoprecipitation assay

HEK-293FT cells were seeded in 10 cm dish at a density of 5 × 10^6^ per dish. 24 h later, plasmids were transfected into HEK-293FT cells by Polythyleneimine transfection buffer (23966-2, Polysciences). 48 h later, cells were lysed with lysis buffer (20 mM Hepes pH 8.0, 0.2 mM EDTA, 5% glycerol, 150 mM NaCl, 1% NP-40). The lysates were incubated on ice for 30 min and sonicated at 10% duty for 10 s on ice with 35% amplitude followed by centrifugation for 15 min at 13,500 rpm at 4 °C. Equal amounts of supernatants were immunoprecipitated with anti-GFP (50430-2-AP, Proteintech) or FLAG antibody, and protein A/G-sepharose beads overnight at 4 °C. Then the beads were washed 3 times before boiled with loading buffer.

### DNA repair assays and flow cytometry

EJ5-U2OS cells were transfected with 5 μg of pLVX-SMYD3-3 × FLAG, or 30 μl siRNAs, 5 μg of I-SceI, and 15 ng DsRed on a Lonza 4D machine (DT-130 program). Then cells were incubated in the cultured medium for 72 h before FACS analysis on flow cytometry (Accuri C6, BD Biosciences). For each sample, 1 × 10^5^ cells were counted. The repair efficiency was calculated as the ratio of GFP-positive cells over the DsRed-positive cells. Each experiment was repeated at least 3 times in triplicate.

### Cell Counting Kit-8 (CCK-8) assay

HEC1B and ISK cells were seeded in 96-well plates at a density of 100 cells per well. At 24, 48, 72, 96, and 120 h, 10 μl of Cell Counting Kit-8 (HY-K0301, MCE) assay reagent in 100 μl of the cultured medium was added into each well. After 2 h, absorbance was examined at 450 nm using a SpectraMax 190 microplate reader (Molecular Devices, Sunnyvale).

### Colony formation assay

1 × 10^3^ cells were plated in each well of 6-well plates. 24 h later, cells were exposed to radiation or without treatment. After incubation for 14 days at 37 °C, colonies were stained with 0.5% crystal violet and counted using ImageJ software.

### Migration and invasion assay

Cell migration was determined by using the Transwell (351157, Corning) migration assay. HEC1B and ISK cells were seeded in a serum-free medium on the upper chambers at a density of 5 × 10^4^ cells per well. The lower chambers were filled with cultured medium. After incubation for 16 h, each chamber was stained with AM (C3100MP, Thermo Fisher Scientific). The migrated cells were imaged by fluorescence analysis (Nikon, Japan). Cell invasion was determined by using the Transwell (3422, Corning) invasion assay. HEC1B and ISK cells were seeded into the upper chambers covered with Matrigel (356234, Corning) for 16 h. The invasive cells were stained with 0.5% crystal violet and counted using ImageJ software.

### Tumor growth in vivo

The animal study was approved by the Ethics Review Committee for Animal Experimentation of Tongji University. Nude mice (female, 4 weeks) were housed in our institutional pathogen-free mouse facilities. HEC1B (1 × 10^6^) cells were suspended in 100 μl of PBS buffer and injected into the flanks of the nude mice. At the end of 3- weeks, the mice were sacrificed, and the tumor xenografts were collected and weighed. For the spleen injection model, HEC1B (1 × 10^5^) cells were suspended in 40 μl of PBS buffer and injected into the spleen of the nude mice. At the end of 8 weeks, the mice were sacrificed, and the livers were collected and fixed in paraformaldehyde for HE and IHC. For the in vivo pharmaceutical test, HEC1B (1 × 10^6^) cells were suspended in 100 μl of PBS buffer and injected into the flanks of the nude mice. At 3 weeks of incubation, 50 μL of BCI-121 (50 μM) were administrated intratumorally twice a week. 2 Gy of γ-ray was pretreated to cells in IR group and BCI-121 + IR group. The tumors were harvested at the end of 4 weeks.

### Tumor tissue samples and IHC

The endometrial cancer samples were obtained from patients who underwent surgical treatment at the Shanghai First Maternity and Infant Hospital. Fresh EC tissues (*n* = 10) and tumor-adjacent tissues (*n* = 10) were collected for qRT-PCR analyses. Paraffin-embedded tissues (endometrial tumor = 15, para-tumor = 3) were obtained for IHC. The study was approved by the Ethics Committee of the hospital. Paraffin-embedded Endometrial cancer tissue microarray (TMA) were purchased from Shanghai OUTDO BIOTECH (HUteA045PG01, OUTDO BIOTECH). Slides were deparaffinized in dimethylbenzene twice for 15 min, followed by rehydrating in 100%-85%-75% ethanol and immersing in 3% H_2_O_2_ for 10 min at room temperature. Antigens were retrieved in citric buffer (G1201-1L, Servicebio) at 97 °C for 30 min, and blocked with blocking buffer (P0103, Beyotime) before cooled down for 1 h. The slides were then stained with anti-SMYD3 (1:100, 12011-1-AP, Proteintech). Tissue samples from the xenograft model were stained with additional anti-Ki67 (1:100, GB111141, Servicebio) and anti-Caspase-3 (1:100, GB11532, Servicebio). The secondary antibody and DAB staining solution were from the MaxVision II kit (MXB Biotechnologies, Fuzhou). At last, counterstained slides with hematoxylin and dehydrated with 75%-85%-100% ethanol. Each TMA spot was measured by both the intensity and the percentage of positive cells. Intensity: 1. low; 2. weak; 3. moderate; 4. strong. Percentage of positive cells: 1. 0–25%; 2. 25–50%; 3. 50–75%; 4. 75–100%. Final score = intensity*percentage.

### Statistical analysis

Data are shown as the mean ± SD for the experiments repeated with at least 3 replicates. For parametric data comparisons, *t* test, one-way ANOVA, and two-way ANOVA were conducted. All statistical analyses and graphs were generated using GraphPad Prism (v9.2.0, La Jolla, USA). A *P* < 0.05 was considered statically significant (not significant, *P* > 0.05, **P < 0.01, ***P < 0.001, ****P < 0.0001).

## Results

### SMYD3 is upregulated in endometrial cancer and associated with tumor progression

**Fig. 1:**
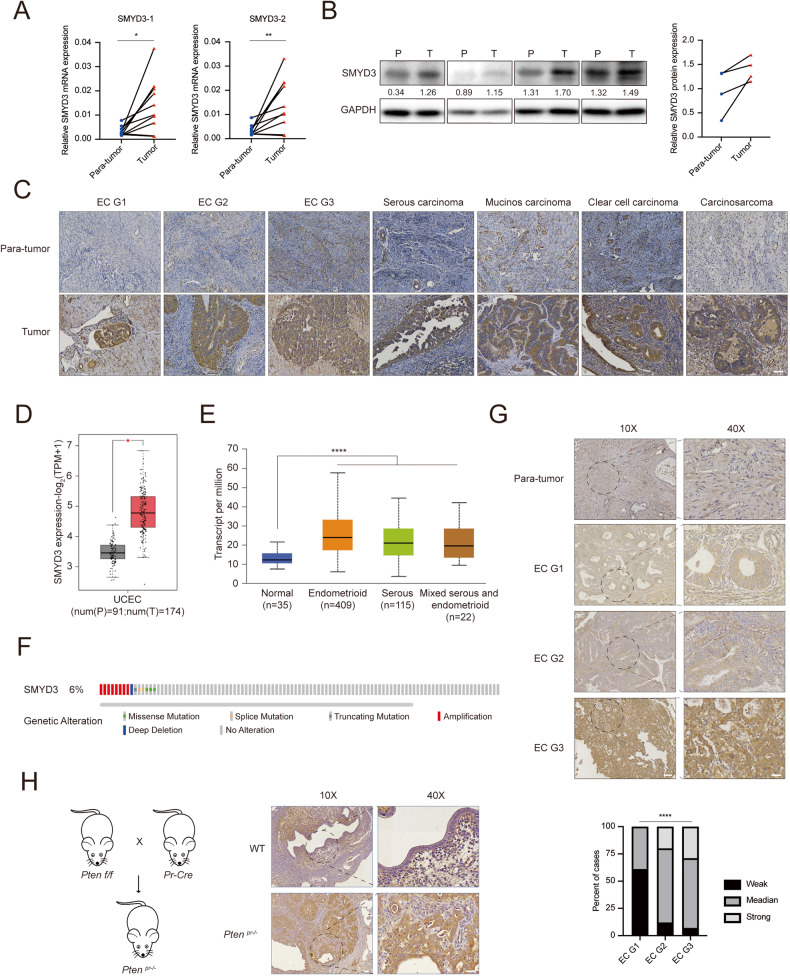
SMYD3 is upregulated in endometrial cancer and associated with tumor progression. **A**, **B** The SMYD3 expression levels in para-tumor and tumor of EC tissues were measured by qRT-PCR (**A**) and Western blot (**B**), SMYD3-1, SMYD3-2 represented two different primers for human SMYD3. *n* = 10 for qRT-PCR **C** Immunohistochemistry of SMYD3 expression in para-tumor and tumor of EC tissues at different pathologic categories; scale bar, 60 μm. **D** The relative mRNA expression of SMYD3 in normal endometrial tissues and endometrial cancer tissues from the TCGA cohort using GEPIA 2 (http://gepia2.cancer-pku.cn/). **E** SMYD3 expression levels in different histological subtypes of EC with data from the TCGA database using UALCAN (http://ualcan.path.uab.edu/). **F** SMYD3 alterations in patients with EC tissues in the TCGA database using cBioPortal (https://www.cbioportal.org/). **G** Immunohistochemistry tissue microarrays showed SMYD3 expression level in para-tumor tissue and EC G1-G3 tissues. *n* = 18 for EC G1, *n* = 25 for EC G2, *n* = 14 for EC G3. Scale bar, 100 μm for ×10; 30 μm for ×40. **H** Immunohistochemistry of SMYD3 expression in the uteri of 30-week-old *Pten* knockout and *Pten* wild-type mice. Scale bar, 100 μm for ×10; 30 μm for ×40.

To assess the role of SMYD3 in endometrial cancer, we first collected fresh paired samples from patients with EC. Both mRNA and protein levels of SMYD3 were elevated in EC tissues compared to adjacent tissues (Fig. 1A, B). Immunohistochemistry (IHC) staining of human endometrial cancer tissues across various pathological categories further underscored the significant upregulation of SMYD3 in different EC subtypes (Fig. 1C). Subsequently, we conducted an analysis of SMYD3 expression in The Cancer Genome Atlas (TCGA) database via several websites for public analysis. The analysis revealed that the expression level of SMYD3 was significantly increased in EC tissues compared with normal controls, as well as endometrioid, serous carcinoma or mixed serous and endometrioid (Fig. 1D, E). Additionally, SMYD3 amplification was observed in 6% of EC patients (Fig. 1F). To further establish the clinical significance of heightened SMYD3 expression, we examined endometrial cancer tissue microarrays, revealing a clear correlation between SMYD3 expression levels and EC clinical grade (Fig. 1G). Considering that *Pten* is the most common hotspot genetic mutated gene in human endometrial cancer, its inactivation is a common event in EC pathogenesis. We utilized a *Pten* knockout genetic engineering mouse model, a widely employed model for studying endometrial cancer [38]. We crossed *Pten* (*Pten*^*loxP/loxP*^) floxed mice with *Pr*^*cre/+*^ mice and obtained the uterus for immunohistochemistry. The expression of Smyd3 in mouse endometrial carcinoma was significantly upregulated compared with that in normal uterus (Fig. 1H). Altogether, these findings indicate that SMYD3 contributes to endometrial cancer progression.

### SMYD3 enhances endometrial cancer cell proliferation, migration, and invasion abilities

**Fig. 2:**
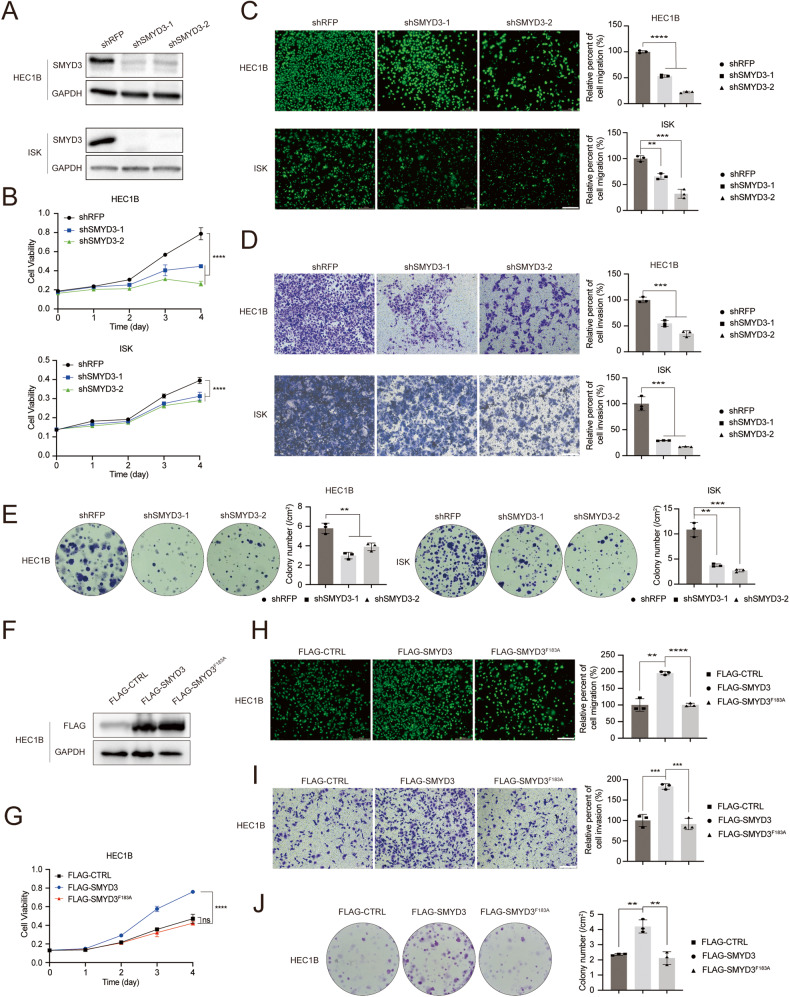
SMYD3 promotes the abilities of cell proliferation, migration, invasion, and colony formation in EC cell lines in vitro. **A** Western blotting of the indicated proteins in whole cell lysates (WCLs) from HEC1B cells and Ishikawa cells stably expressing shRFP, shSMYD3-1, or shSMYD3-2. **B** CCK-8 cell proliferation analysis of HEC1B cells and ISK cells stably expressing shRFP, shSMYD3-1, or shSMYD3-2. Data are shown as the mean ± SD (*n* = 3). **C**, **D** Left graph, cell migration analysis (**C**), and invasion analysis (**D**) of HEC1B cells and ISK cells stably expressing shRFP, shSMYD3-1, or shSMYD3-2. Right graph, the quantitative data are shown as the mean ± SD (*n* = 3). Scale bar, 200 μm. **E** Colony formation assay of HEC1B cells and ISK cells stably expressing shRFP, shSMYD3-1, or shSMYD3-2. **F** Western blotting of the indicated proteins in WCLs from HEC1B cells stably expressing FLAG-CTRL, FLAG-SMYD3, or FLAG-SMYD3^F183A^. **G** CCK-8 cell proliferation analysis of HEC1B cells stably expressing FLAG-CTRL, FLAG-SMYD3, or FLAG-SMYD3^F183A^. Data are shown as the mean ± SD (*n* = 3). **H**, **I** Left graph, cell migration analysis (**H**), and cell invasion analysis (**I**) of HEC1B cells stably expressing FLAG-CTRL, FLAG-SMYD3, or FLAG-SMYD3^F183A^. Right graph, the quantitative data are shown as the mean ± SD (*n* = 3). Scale bar, 200 μm. **J**. Colony formation assay of HEC1B cells stably expressing FLAG-CTRL, FLAG-SMYD3, or FLAG-SMYD3^F183A^.

The observations above prompted us to explore the potential biological function of SMYD3 in endometrial cancer progression. First, we investigated the effects of SMYD3 on cancer cell proliferation by a loss-of-function assay. The EC cell lines HEC1B and Ishikawa (ISK) were stably transfected with short hairpin RNA (shRNA) targeting SMYD3 and its corresponding negative control (shRFP). The efficacy of shRNA inhibition was validated post-puromycin selection (Fig. 2A). Subsequent Cell Counting Kit-8 (CCK-8) assays unveiled a significant reduction in cell proliferation upon SMYD3 depletion in HEC1B and ISK cells (Fig. 2B). Moreover, we investigated the impact of SMYD3 on cell migration and invasion using Transwell and Matrigel invasion assays. Results indicated a noteworthy decrease in cell migration and invasion abilities following SMYD3 downregulation in both the HEC1B and ISK cell lines (Fig. 2C, D). To further elucidate the role of SMYD3 in cell growth, a colony formation assay was performed, revealing fewer and smaller colonies in the SMYD3 downregulated group compared to the control group, signifying a suppressive effect on EC cell proliferation (Fig. 2E).

Consistent with the inhibitory effects observed, ectopic expression of SMYD3 in HEC1B cells bolstered proliferation, migration, invasion, and colony formation abilities. Notably, the enzymatically inactive SMYD3-F183A failed to induce these effects (Fig. 2F–J). Collectively, these results underscore the oncogenic properties of SMYD3, demonstrating its capability to promote proliferation, migration, and invasion in EC cells, contingent upon its methyltransferase activity.

### SMYD3 drives tumorigenicity and metastasis of endometrial cancer in vivo

**Fig. 3:**
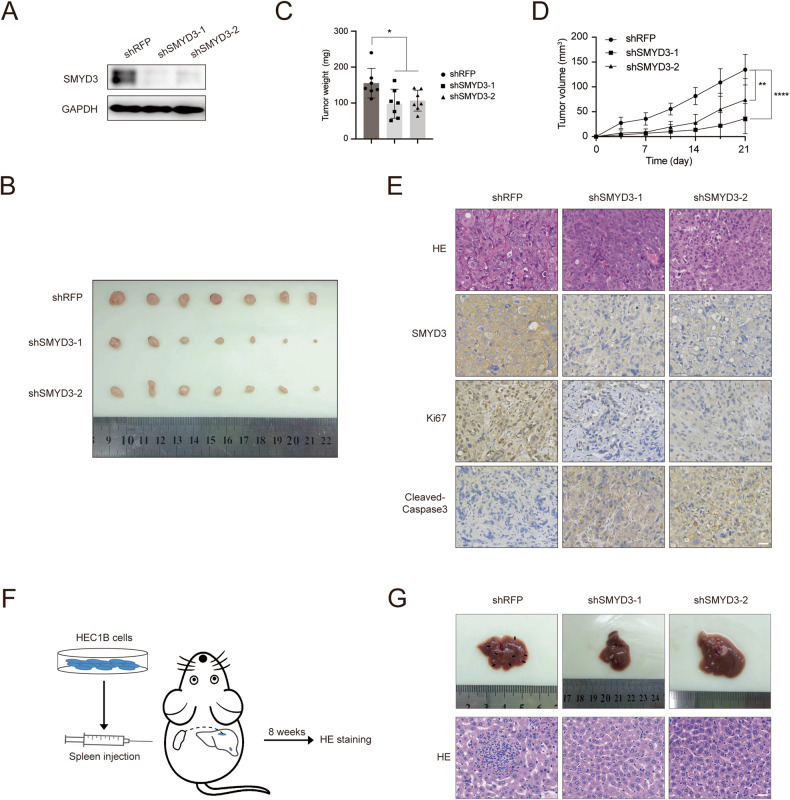
Knockdown of SMYD3 impairs tumorigenicity and metastasis of EC in vivo. **A** Western blotting of the indicated proteins in WCLs from HEC1B cells stably expressing shRFP, shSMYD3-1, or shSMYD3-2. **B** Images of HEC1B cells stably expressing shRFP, shSMYD3-1, or shSMYD3-2-implanted tumors from nude mice. **C** The weight of xenograft tumors shown in **B**. *n* = 7. **D** The tumor volume of xenograft tumors was measured on the indicated days after injection. **E** Representative images of immunofluorescence staining of SMYD3, Ki67, and cleaved-Caspase3. Scale bar, 30 μm. **F** Schematic diagram of cell inoculation and liver metastases in nude mice. **G** Images of HEC1B cells stably expressing shRFP, shSMYD3-1, or shSMYD3-2 liver metastases from nude mice (Upper graph). HE staining of HEC1B cells stably expressing shRFP, shSMYD3-1, or shSMYD3-2 liver metastases from nude mice (Lower graph). Scale bar, 30 μm.

To further investigate whether SMYD3 plays the same oncogenic role in vivo, we injected HEC1B cells (shRFP, shSMYD3-1, shSMYD3-2) into the flanks of female nude mice. Tumors derived from the shSMYD3 groups exhibited markedly lower weights and volumes compared to those originating from the shRFP-HEC1B injection group (Fig. 3A–D). Consistent with the reduction in tumor size, SMYD3-deficient tumor tissues exhibited diminished proliferation (Ki67) and increased cell death (cleaved-Caspase-3), as revealed by immunohistochemical staining (Fig. 3E). Moreover, we examined the impact of SMYD3 on liver metastasis by implanting the spleens of mice, following a paradigm established in previous endometrial cancer studies [39, 40]. The shRFP and shSMYD3-HEC1B stable cell lines were introduced into the spleens of nude mice, and tissue samples were collected at approximately 8 weeks. As expected, most mice from the shRFP group developed liver metastases, in contrast to the SMYD3-deficient group. The clone numbers of metastatic tumor burden in the liver from the SMYD3 knockdown group were much lower compared with the control group, and the size of metastasis clone size showed a similar trend (Fig. 3F, G). In summary, these findings are consistent with the in vitro results, indicating that SMYD3 promotes endometrial cancer growth and metastasis in vivo.

### SMYD3 is recruited to DNA damage sites in a PARP1-dependent manner

**Fig. 4:**
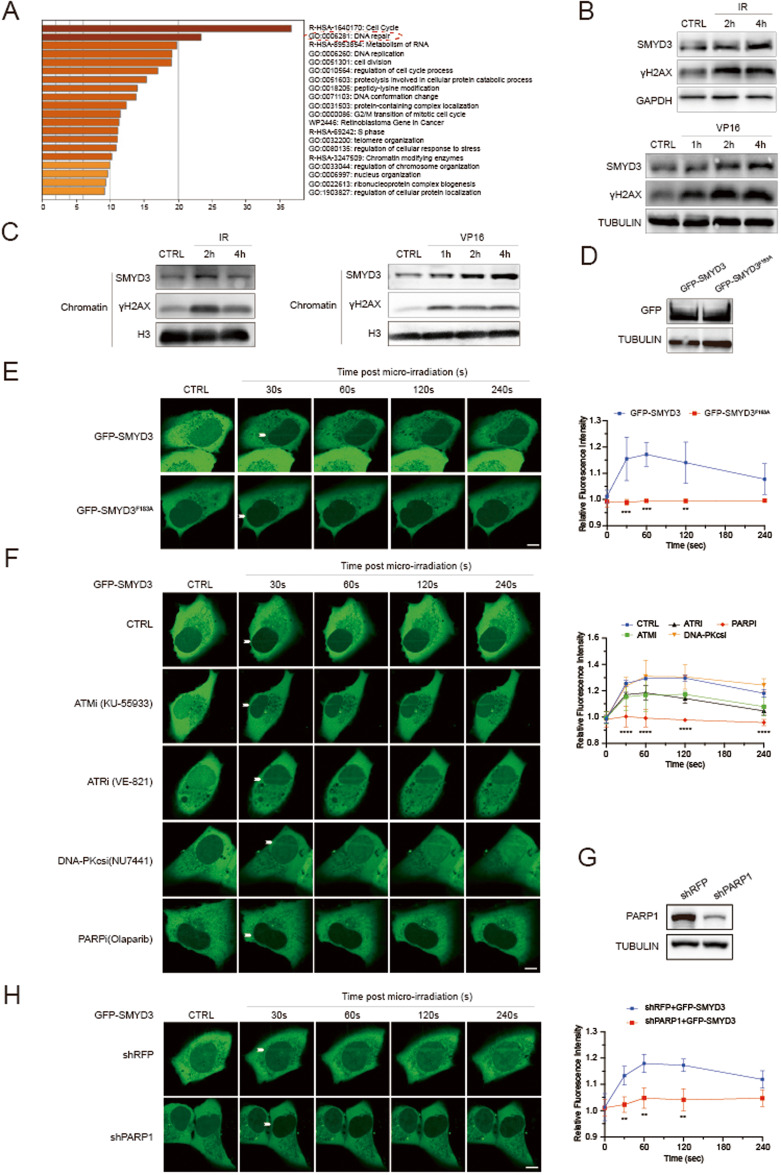
SMYD3 is recruited to DNA damage sites in a PARP1-dependent manner. **A** The top upregulated Gene Ontology (GO) terms in the enrichment analysis. **B** Western blotting of the indicated proteins in WCLs from HEC1B cells after γ-ray irradiation at 10 Gy (Left graph). Western blotting of the indicated proteins in WCLs treated with 40 μM VP16 for 1, 2, and 4 h (Right graph). **C** Western blotting of chromatin proteins extracted from HEC1B cells after γ-ray irradiation at 10 Gy (Left graph). Western blotting of chromatin proteins extracted from HEC1B cells treated with 40 μM VP16 for 1, 2, and 4 h (Right graph). **D** Western blotting of the indicated proteins in WCLs from U2OS cells transfected with indicated plasmids. **E** Dynamics of GFP-SMYD3 or GFP-SMYD3^F183A^ accumulation at micro-irradiated sites (Left graph). Relative fluorescence intensity of GFP-SMYD3 and GFP-SMYD3^F183A^ at micro-irradiated sites in the experiments described above (Right graph). Scale bar, 5 μm. **F** Dynamics of GFP-SMYD3 accumulation at micro-irradiated sites treated with different inhibitors at the indicated concentration for 24 h (Left graph). Relative fluorescence intensity of GFP-SMYD3 at micro-irradiated sites in the experiments described in the left graph (Right graph). Scale bar, 5 μm. **G** Western blotting of the indicated proteins in WCLs from U2OS cells stably expressing shRFP, shPARP1. **H** Time-lapse images of GFP-SMYD3 accumulation at micro-irradiated sites in U2OS cells stably expressing shRFP or shPARP1 (Left graph). Relative fluorescence intensity of GFP-SMYD3 at micro-irradiated sites in the experiments described in the left graph (Right graph). Scale bar, 5 μm.

To further elucidate the molecular mechanisms underlying the effects of SMYD3 in endometrial cancer, we used pathway analysis (http://metascape.org/) with patient samples from the TCGA database to identify SMYD3-correlated genes and pathways. As shown in Fig. 4A, SMYD3 was closely associated with the DNA repair pathway. To investigate whether SMYD3 is involved in the DNA damage response, we initially examined the effect of DNA damage on the total protein level and cellular localization of SMYD3. Intriguingly, we observed a significant increase in the total protein level of SMYD3 upon ionizing radiation (IR) or treatment with the chemotherapeutic compound VP16 (Fig. 4B). Furthermore, the elevated SMYD3 was predominantly localized in the chromatin-bound fraction, as determined through detergent and chromatin fractionation methods (Fig. 4C). To examine the dynamic enrichment of SMYD3 at sites of DNA damage, we transfected GFP-SMYD3 and its enzymatically dead GFP-SMYD3-F183A plasmids into U2OS cells (Fig. 4D). Time-lapse imaging showed that laser micro-irradiation [41] with a 365 nm UV laser led to a rapid accumulation (~30 s) of GFP-SMYD3 at sites of DNA damage, as shown by the arrow (Fig. 4E). In addition, the enzymatically dead GFP-SMYD3-F183A abolished this dramatic recruitment, suggesting that the methylation activity of SMYD3 was essential for its recruitment to DNA damage sites (Fig. 4E). To identify the key DDR proteins regulating the recruitment of SMYD3 to DNA damage sites, we employed four different inhibitors targeting various steps of DNA damage repair. The recruitment of SMYD3 to DNA damage sites was specifically impaired when cells were treated with the PARP inhibitor Olaparib. Conversely, the ATM inhibitor KU-55933, the ATR inhibitor VE-821, and the DNA-PKcs inhibitor NU7441 had no effect on blocking this recruitment (Fig. 4F). Moreover, consistent with the impact of PARP inhibitors, knockdown of PARP1 also abolished the recruitment of SMYD3 to DNA damage sites (Fig. 4G, H). These findings collectively demonstrate that SMYD3 is recruited to chromatin in response to DNA damage through a mechanism involving PARP1.

### SMYD3 promotes NHEJ repair via interaction with NHEJ proteins and modulation of LIG4/XRCC4/XLF dynamics at DNA damage sites

**Fig. 5:**
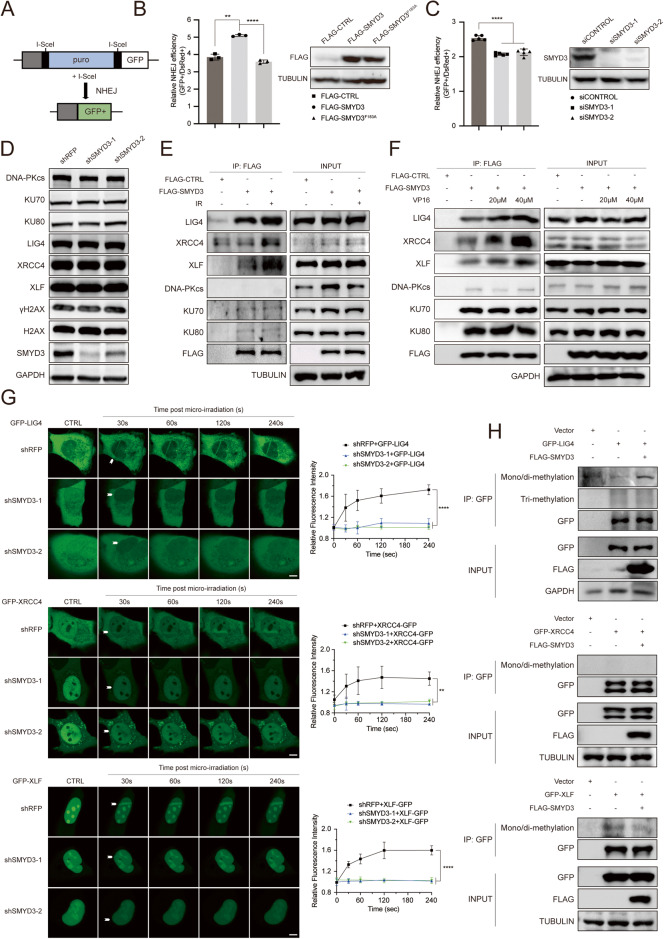
SMYD3 promotes NHEJ repair via interaction with NHEJ proteins and modulates LIG4/XRCC4/XLF dynamics at DNA damage sites. **A** Schematic diagram of NHEJ fluorescent reporter repair in EJ5-U2OS cells. Two I-SceI sites were constructed between the whole GFP sites. Once transfected with I-SceI, cells will emit green fluorescence when repaired successfully through NHEJ pathway. **B**, **C** Flow cytometry analysis of GFP^+^ and DsRed^+^ cells of EJ5-U2OS cells transfected with I-SceI and indicated plasmids or siRNAs. The overexpression or knockdown efficiency of SMYD3 were determined by western blot as shown in the right graph. **D** Western blotting of the indicated proteins in WCLs from HEC1B cells stably expressing shRFP, shSMYD3-1, or shSMYD3-2. **E**, **F** Co-IP analysis of the indicated proteins in WCLs from HEK-293FT cells transfected with FLAG-SMYD3 treated with 10 Gy γ-ray irradiation (**E**) or VP16 at indicated concentration (**F**). **G** Dynamics of GFP-LIG4, GFP-XRCC4 or GFP-XLF accumulation at micro-irradiated sites in U2OS cells stably expressing shRFP, shSMYD3-1 or shSMYD3-2 (Left graph). Relative fluorescence intensity of GFP-LIG4, GFP-XRCC4 or GFP-XLF at micro-irradiated sites in the experiments described in the left graph (Right graph). Scale bar, 5 μm. **H** IP analysis of the mono/di-methylation or tri-methylation of the indicated proteins in WCLs from HEK-293FT cells transfected with GFP-LIG4, GFP-XRCC4, GFP-XLF or FLAG-SMYD3.

DNA double-strand breaks (DSBs) represent a critical form of DNA damage, primarily repaired by the HR and NHEJ pathways. While previous studies have established that SMYD3 can directly interact with key members involved in the HR pathway, its potential role in the NHEJ pathway remained unclear. To address this, we introduced SMYD3 into EJ5-U2OS cells, a well-established cell line with an NHEJ reporter cassette, to evaluate the impact of SMYD3 on NHEJ repair efficiency. The results demonstrated that the wild-type SMYD3 plasmid, but not the enzymatically dead F183A-SMYD3 plasmid, significantly increased NHEJ repair efficiency (Fig. 5A, B). Conversely, when SMYD3 was depleted using two independent siRNAs, a reduction in NHEJ repair efficiency was observed (Fig. 5C), highlighting the role of SMYD3 and its methyltransferase activity in NHEJ repair. To identify potential partners associated with SMYD3-mediated NHEJ repair, we assessed key DNA repair proteins in the NHEJ pathway, including DNA-PKcs, KU70, KU80, LIG4, XRCC4, and XLF. However, the expression levels of these proteins remained unchanged upon SMYD3 knockdown (Fig. 5D). Further investigations using coimmunoprecipitation assays revealed an increased interaction between SMYD3 and the LIG4/XRCC4/XLF complex upon IR/VP16 treatment, whereas no significant changes were observed in the KU70/KU80/DNA-PKcs complex (Fig. 5E, F). Moreover, in a laser micro-irradiation assay, impaired recruitment of the LIG4/XRCC4/XLF complex was observed in SMYD3-depleted cells (Fig. 5G). The investigation into direct methylation targets revealed that in the presence of SMYD3, the methylation level of LIG4 significantly increased, while no methylation signals were detected from XRCC4 or XLF proteins (Fig. 5H). These findings collectively demonstrate that SMYD3 directly methylates LIG4, influencing the interaction and recruitment of the LIG4/XRCC4/XLF complex and ultimately contributing to efficient NHEJ repair.

### Pharmacological intervention of SMYD3 enhances the response of EC cells to radiotherapy

**Fig. 6:**
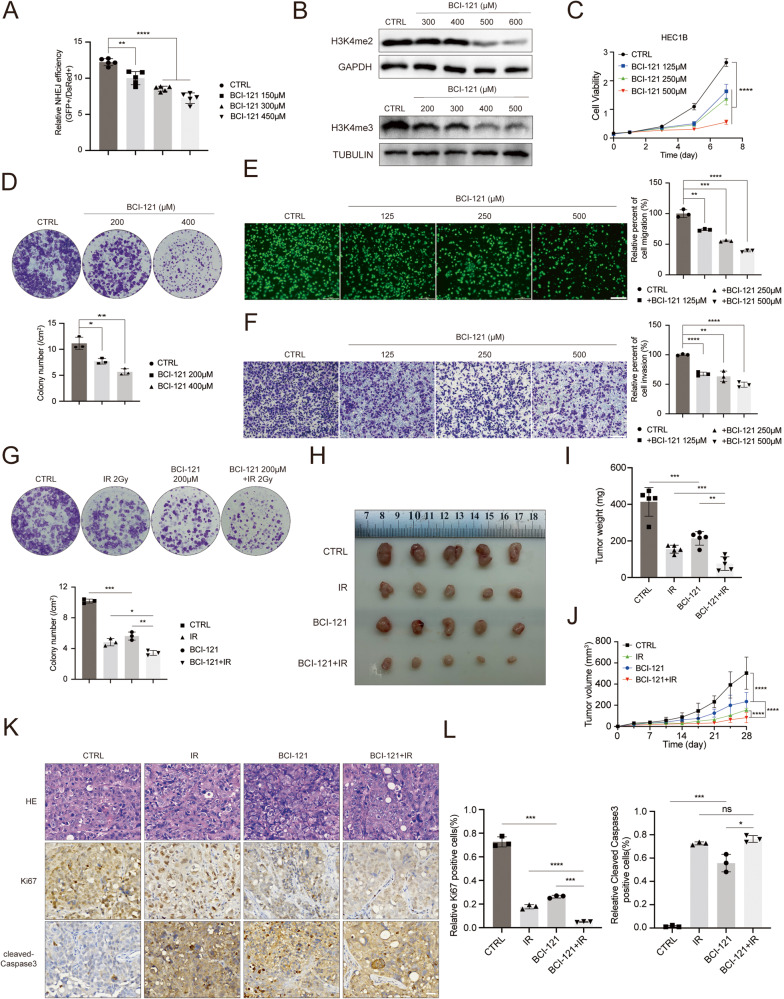
Pharmacological intervention of SMYD3 enhances the response of EC cells to radiotherapy. **A** Flow cytometry analysis of GFP^+^ and DsRed^+^ cells of EJ5-U2OS cells upon BCI-121 treatment. **B** Western blotting of the indicated proteins in WCLs from HEC1B cells upon treatment with BCI-121. **C** CCK-8 cell proliferation analysis of HEC1B cells upon BCI-121 treatment. **D** Colony formation assay of HEC1B cells upon BCI-121 addition. **E**, **F** Left graph, cell migration analysis (**E**), and cell invasion analysis (**F**) of HEC1B cells upon BCI-121 treatment. Right graph, the quantitative data are shown as the mean ± SD (*n* = 3). Scale bar, 200 μm. **G** Images of HEC1B cells upon either BCI-121 treatment irradiation or combined treatments. **H** Images of the tumors from HEC1B cells treated with BCI-121 (500 μM) or irradiation or combined treatment. **I** The weight of xenograft tumors shown in **G**. *n* = 5. **J** The tumor volume of xenograft tumors was measured on the indicated days after injection. **K** Representative images of immunofluorescence staining of the expression of Ki67 and cleaved-Caspase3. Scale bar, 30 μm. **L** The quantitative IHC data shown in **K**.

BCI-121 is regarded as a specific and effective inhibitor of SMYD3 [42]. To address whether targeting SMYD3 is a translational strategy to inhibit EC progression, we used BCI-121 for in vitro and in vivo assays. Employing EJ5-U2OS reporter cells, we evaluated the NHEJ repair efficiency upon BCI-121 treatment, revealing a significant dose-dependent inhibition of NHEJ repair (Fig. 6A). Western blotting assays showed that the H3K4me2 and H3K4me3 expression levels decreased upon increasing the dose of BCI-121 in HEC1B cells as positive controls for detecting the effect of the inhibitor [42] (Fig. 6B). As expected, the proliferation ability and viability of HEC1B cells were significantly inhibited upon BCI-121 treatment, as examined by CCK-8 assays and colony formation assays [26, 43–51] (Fig. 6C, D). Transwell assays also demonstrated that EC cells treated with BCI-121 had a much lower migration and invasion ability than control cells (Fig. 6E, F). Given the association between SMYD3 and DNA repair, we explored SMYD3’s role in radiosensitivity. The combination of the SMYD3 inhibitor BCI-121 with radiation resulted in a significant synergistic effect, notably inhibiting the survival of EC cells (Fig. 6G). In an in vivo xenograft model, intra-tumoral injection of BCI-121 or radiation alone moderately repressed tumor growth compared to the control group. However, the combination therapy of BCI-121 and radiation led to a remarkable reduction in tumor size (Fig. 6H–J). Consistent with reduced tumor size, immunohistochemical staining of Ki67 showed decreased proliferation, while enhanced cleaved-caspase-3 staining indicated increased apoptosis in the combination treatment mouse tissues (Fig. 6K, L). These results, in agreement with our cell-based findings, indicate that pharmacological intervention targeting SMYD3 as a radiosensitizer holds promise for an enhanced therapeutic outcome in EC. Overall, these findings suggest that SMYD3 could indeed serve as a potential therapeutic target in endometrial cancer, offering insights into a promising treatment strategy.

## Discussion

In this study, we have uncovered a pivotal oncogenic role of SMYD3 in the progression of EC, highlighting its potential as a therapeutic target through the NHEJ repair pathway. Our comprehensive analysis reveals a significant upregulation of SMYD3 in EC patient samples, correlating with cancer progression. Through pathway analysis, we introduce a novel mechanism whereby SMYD3 regulates NHEJ repair by methylating LIG4, influencing the recruitment of the LIG4/XRCC4/XLF complex.

As a SET domain-containing methyltransferase, SMYD3 has been implicated in various cellular functions and is upregulated in several cancers. Our study extends this understanding to endometrial cancer, demonstrating that the knockdown of SMYD3 hinders cell proliferation, migration, and invasion abilities in EC cells. In vivo, mouse models further validate the role of SMYD3 in reducing both tumor growth and metastasis. Importantly, we provide evidence that the oncogenic properties of SMYD3 in endometrial cancer depend on its methyltransferase activity, as demonstrated by using an enzymatically inactive SMYD3-F183A plasmid.

Pathway analysis identifies a close association between SMYD3 and the DNA repair pathway in endometrial cancer. In response to DNA damage, SMYD3 is recruited to damage sites in a PARP1-dependent manner. We reveal an enhanced interaction between SMYD3 and the LIG4/XRCC4/XLF complex upon DNA damage, leading to specific methylation of LIG4. This methylation promotes the recruitment of the LIG4/XRCC4/XLF complex, facilitating efficient NHEJ repair. Our study addresses the gap in understanding SMYD3’s role in the NHEJ repair pathway, which is critical for DNA double-strand break repair.

While the study demonstrates the potential of SMYD3 as a therapeutic target, there are considerations about the high concentrations of the SMYD3 inhibitor BCI-121. The requirement for high concentrations may induce off-target effects, emphasizing the need for more efficacious SMYD3 inhibitors. Additionally, the methylation sites of LIG4 by SMYD3 remain unknown, presenting an avenue for further exploration. The specific mutation forms of LIG4 resulting from these methylation sites also warrant investigation.

In conclusion, this study unveils a novel mechanism by which SMYD3 contributes to endometrial cancer, shedding light on its dynamic regulation of the LIG4/XRCC4/XLF complex in the NHEJ pathway. Targeting SMYD3, particularly in combination with radio/chemotherapy, emerges as a promising strategy for anticancer therapy in endometrial cancer. Further research is needed to refine the use of SMYD3 inhibitors and explore the detailed relationship between SMYD3 and LIG4, offering potential avenues for clinical application and deeper mechanistic understanding.

## Data Availability

All the data and techniques within the article and Supplementary Information file are available from the corresponding author upon reasonable request.
